# Supplementary material for: Non-autistic persons modulate their speech rhythm while talking to autistic individuals
Source: PLoS One. 2023 Sep 28;18(9):e0285591. doi: 10.1371/journal.pone.0285591 (PMC10538692; doi:10.1371/journal.pone.0285591)
Supplement: S1 Appendix — (DOC) [file pone.0285591.s001.doc]

**Statistical results of ASD speech and TD speech**

**・FFT analyses.**

**Within Subjects Effects**

|  | **Sum of Squares** | **df** | **Mean Square** | **F** | **p** | **η²_p_** |
| --- | --- | --- | --- | --- | --- | --- |
| Rhythm | 0.0540 | 1.20 | 0.0451 | 493.69 | < .001 | 0.759 |
| Rhythm ┯ Group | 7.74e-4 | 1.20 | 6.47e-4 | 7.08 | 0.006 | 0.043 |
| Residual | 0.0172 | 187.83 | 9.14e-5 |  |  |  |


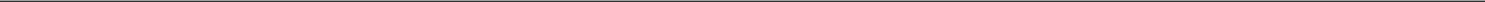


* = Significance based on False Discovery Rate (FDR), SE = Standard error, df = degrees of freedom

**Between Subjects Effects**

|  | **Sum of Squares** | **df** | | **Mean Square** | **F** | **p** | **η²_p_** |
| --- | --- | --- | --- | --- | --- | --- | --- |
| Group | 8.48e-4 | 1 | 8.48e-4 | | 3.31 | 0.071 | 0.021 |
| Residual | 0.0403 | 157 | 2.57e-4 | |  |  |  |

* = Significance based on False Discovery Rate (FDR), SE = Standard error, df = degrees of freedom

**Post Hoc Comparisons - Rhythm**

| **Rhythm** |  | **Rhythm** | **Mean Difference** | **SE** | **df** | **t** | **p (FDR)** |
| --- | --- | --- | --- | --- | --- | --- | --- |
| prosody | - | syllable | 0.00908 | 8.33e-4 | 314 | 10.9 | < .001* |
|  | - | phoneme | 0.02580 | 8.33e-4 | 314 | 31.0 | < .001* |
| syllable | - | phoneme | 0.01672 | 8.33e-4 | 314 | 20.1 | < .001* |

* = Significance based on False Discovery Rate (FDR), SE = Standard error, df = degrees of freedom

**Post Hoc Comparisons - Rhythm** ┯ **Group**

| **Rhythm** | **Group** | |  | **Rhythm** | **Group** | **MeanDifference** | **SE** | **df** | **t** | **p(FDR)** |
| --- | --- | --- | --- | --- | --- | --- | --- | --- | --- | --- |
| prosody | | ASD | - | prosody | TD | -0.00603 | 0.00176 | 293 | -3.426 | <.001* |
|  | |  | - | syllable | ASD | 0.00716 | 0.00112 | 314 | 6.390 | <.001* |
|  | |  | - | phoneme | ASD | 0.02269 | 0.00112 | 314 | 20.245 | <.001* |
|  | | TD | - | syllable | TD | 0.01100 | 0.00123 | 314 | 8.927 | <.001* |
|  | |  | - | phoneme | TD | 0.02890 | 0.00123 | 314 | 23.456 | <.001* |
| syllable | | ASD | - | syllable | TD | -0.00219 | 0.00176 | 293 | -1.245 | 0.214 |
|  | |  | - | phoneme | ASD | 0.01553 | 0.00112 | 314 | 13.854 | <.001* |
|  | | TD | - | phoneme | TD | 0.01790 | 0.00123 | 314 | 14.529 | <.001* |
| phoneme | | ASD | - | phoneme | TD | 1.82e-4 | 0.00176 | 293 | 0.103 | 0.918 |

**
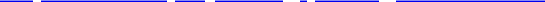
**

* = Significance based on False Discovery Rate (FDR), SE = Standard error, df = degrees of freedom

**・Transfer Entropy analyses.**

**Within Subjects Effects**

|  | **Sum of Squares** | **df** | **Mean Square** | **F** | **p** | **η²p** |
| --- | --- | --- | --- | --- | --- | --- |
| Rhythm | 0.41453 | 1.74 | 0.23881 | 326.90 | < .001 | 0.676 |
|  | 0.41453 | 1.74 | 0.23881 | 326.90 | < .001 | 0.676 |
| Rhythm ┯ Group | 0.01632 | 1.74 | 0.00940 | 12.87 | < .001 | 0.076 |
| Residual | 0.19909 | 272.53 | 7.31e-4 |  |  |  |
| Hierarchy | 0.63071 | 1 | 0.63071 | 325.59 | < .001 | 0.675 |
| Hierarchy ┯ Group | 0.00754 | 1 | 0.00754 | 3.89 | 0.050 | 0.024 |
| Residual | 0.30413 | 157 | 0.00194 |  |  |  |
| Rhythm ┯ Hierarchy | 0.07165 | 2 | 0.03582 | 189.35 | < .001 | 0.547 |
| Rhythm ┯ Hierarchy ┯ Group | 8.89e-4 | 2 | 4.44e-4 | 2.35 | 0.097 | 0.015 |
| Residual | 0.05941 | 314 | 1.89e-4 |  |  |  |

* = Significance based on False Discovery Rate (FDR), SE = Standard error, df = degrees of freedom

**Between Subjects Effects**

|  | **Sum of Squares** | **df** | **Mean Square** | **F** | **p** | **η²p** |
| --- | --- | --- | --- | --- | --- | --- |
| Group | 0.0630 | 1 | 0.0630 | 2.95 | 0.088 | 0.018 |
| Residual | 3.3529 | 157 | 0.0214 |  |  |  |

* = Significance based on False Discovery Rate (FDR), SE = Standard error, df = degrees of freedom

**Post Hoc Comparisons – Rhythm**

| **Rhythm** |  | **Rhythm** | **Mean Difference** | **SE** | **df** | **t** | **p(FDR)** |
| --- | --- | --- | --- | --- | --- | --- | --- |
| Pro-Sy | - | Pro-Ph | 0.0249 | 0.00201 | 314 | 12.4 | < .001* |
|  | - | Sy-Ph | -0.0264 | 0.00201 | 314 | -13.1 | < .001* |
| Pro-Ph | - | Sy-Ph | -0.0513 | 0.00201 | 314 | -25.6 | < .001* |

* = Significance based on False Discovery Rate (FDR), SE = Standard error, df = degrees of freedom

**Post Hoc Comparisons - Rhythm** ┯ **Group**

| **Rhythm** | **Group** |  | **Rhythm** | **Group** | **Mean Difference** | **SE** | **df** | **t** | **p(FDR)** |
| --- | --- | --- | --- | --- | --- | --- | --- | --- | --- |
| Pro-Sy | ASD | - | Pro-Sy | TD | -0.00884 | 0.00978 | 176 | -0.903 | 0.368 |
|  |  | - | Pro-Ph | ASD | 0.02661 | 0.00270 | 314 | 9.857 | < .001* |
|  |  | - | Sy-Ph | ASD | -0.01683 | 0.00270 | 314 | -6.236 | < .001* |
|  | TD | - | Pro-Ph | TD | 0.02321 | 0.00297 | 314 | 7.821 | < .001* |
|  |  | - | Sy-Ph | TD | -0.03591 | 0.00297 | 314 | -12.101 | < .001* |
| Pro-Ph | ASD | - | Pro-Ph | TD | -0.01224 | 0.00978 | 176 | -1.251 | 0.213 |
|  |  | - | Sy-Ph | ASD | -0.04344 | 0.00270 | 314 | -16.093 | < .001* |
|  | TD | - | Sy-Ph | TD | -0.05912 | 0.00297 | 314 | -19.922 | < .001* |
| Sy-Ph | ASD | - | Sy-Ph | TD | -0.02792 | 0.00978 | 176 | -2.853 | 0.005* |

* = Significance based on False Discovery Rate (FDR), SE = Standard error, df = degrees of freedom

**Post Hoc Comparisons - Hierarchy**

| **Hierarchy** |  | **Hierarchy** | **Mean Difference** | **SE** | **df** | **t** | **p(FDR)** |
| --- | --- | --- | --- | --- | --- | --- | --- |
| High->Low | - | Low->High | 0.0517 | 0.00286 | 157 | 18.0 | < .001 |

* = Significance based on False Discovery Rate (FDR), SE = Standard error, df = degrees of freedom

**Post Hoc Comparisons - Hierarchy ┯ Group**

| **Hierarchy** | **Group** |  | **Hierarchy** | **Group** | **Mean Difference** | **SE** | **df** | **t** | **p(FDR)** |
| --- | --- | --- | --- | --- | --- | --- | --- | --- | --- |
| High->Low | ASD | - | High->Low | TD | -0.0220 | 0.00993 | 185 | -2.21 | 0.028* |
|  |  | - | Low->High | ASD | 0.0460 | 0.00385 | 157 | 11.94 | < .001* |
|  | TD | - | Low->High | TD | 0.0573 | 0.00424 | 157 | 13.53 | < .001* |
| Low->High | ASD | - | Low->High | TD | -0.0107 | 0.00993 | 185 | -1.08 | 0.283 |

* = Significance based on False Discovery Rate (FDR), SE = Standard error, df = degrees of freedom

**Post Hoc Comparisons - Rhythm ┯ Hierarchy**

| **Rhythm** | **Hierarchy** |  | **Rhythm** | **Hierarchy** | **Mean Difference** | **SE** | **df** | **t** | **p(FDR)** |
| --- | --- | --- | --- | --- | --- | --- | --- | --- | --- |
| Pro-Sy | High->Low | - | Pro-Sy | Low->High | 0.06346 | 0.00313 | 220 | 20.28 | < .001* |
|  |  | - | Pro-Ph | High->Low | 0.02441 | 0.00229 | 486 | 10.68 | < .001* |
|  |  | - | Sy-Ph | High->Low | -0.00816 | 0.00229 | 486 | -3.57 | < .001* |
|  | Low->High | - | Pro-Ph | Low->High | 0.02541 | 0.00229 | 486 | 11.12 | < .001* |
|  |  | - | Sy-Ph | Low->High | -0.04458 | 0.00229 | 486 | -19.51 | < .001* |
| Pro-Ph | High->Low | - | Pro-Ph | Low->High | 0.06446 | 0.00313 | 220 | 20.60 | < .001* |
|  |  | - | Sy-Ph | High->Low | -0.03257 | 0.00229 | 486 | -14.25 | < .001* |
|  | Low->High | - | Sy-Ph | Low->High | -0.06999 | 0.00229 | 486 | -30.62 | < .001* |
| Sy-Ph | High->Low | - | Sy-Ph | Low->High | 0.02704 | 0.00313 | 220 | 8.64 | < .001* |

* = Significance based on False Discovery Rate (FDR), SE = Standard error, df = degrees of freedom
