# Supplementary material for: Non-autistic persons modulate their speech rhythm while talking to autistic individuals
Source: PLoS One. 2023 Sep 28;18(9):e0285591. doi: 10.1371/journal.pone.0285591 (PMC10538692; doi:10.1371/journal.pone.0285591)
Supplement: S2 Appendix — (DOC) [file pone.0285591.s002.doc]

**Statistical results of ASD-directed speech and TD-directed speech**

**・FFT analyses.**

**Within Subjects Effects**

**
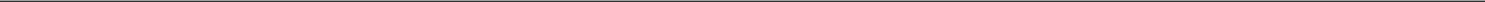
**

|  | **Sum of Squares** | **df** | **Mean Square** | **F** | **p** | **η²p** |
| --- | --- | --- | --- | --- | --- | --- |
| Rhythm | 0.2924 | 1.00 | 0.29150 | 67.64 | < .001 | 0.348 |
| Rhythm ┯ Group | 0.0394 | 1.00 | 0.03932 | 9.12 | 0.003 | 0.067 |
| Residual | 0.5490 | 127.39 | 0.00431 |  |  |  |

* = Significance based on False Discovery Rate (FDR), SE = Standard error, df = degrees of freedom

**Between Subjects Effects**

|  | **Sum of Squares** | **df** | | **Mean Square** | **F** | **p** | **η²_p_** |
| --- | --- | --- | --- | --- | --- | --- | --- |
| Group | 0.0260 | 1 | 0.02600 | | 10.0 | 0.002 | 0.073 |
| Residual | 0.3290 | 127 | 0.00259 | |  |  |  |

* = Significance based on False Discovery Rate (FDR), SE = Standard error, df = degrees of freedom

**Post Hoc Comparisons - Rhythm**

| **Rhythm** |  | **Rhythm** | **Mean Difference** | **SE** | **df** | **t** | **p (FDR)** |
| --- | --- | --- | --- | --- | --- | --- | --- |
| prosody | - | syllable | 0.0453 | 0.00585 | 254 | 7.73 | < .001* |
|  | - | phoneme | 0.0667 | 0.00585 | 254 | 11.39 | < .001* |
| syllable | - | phoneme | 0.0214 | 0.00585 | 254 | 3.66 | < .001* |

* = Significance based on False Discovery Rate (FDR), SE = Standard error, df = degrees of freedom

**Post Hoc Comparisons - Rhythm** ┯ **Group**

| **Rhythm** | **Group** | |  | **Rhythm** | **Group** | **MeanDifference** | **SE** | **df** | **t** | **p(FDR)** |
| --- | --- | --- | --- | --- | --- | --- | --- | --- | --- | --- |
| prosody | | ASD | - | prosody | TD | -0.04539 | 0.00855 | 378 | -5.3118 | < .001* |
|  | |  | - | syllable | ASD | 0.02436 | 0.00764 | 254 | 3.1877 | 0.024 |
|  | |  | - | phoneme | ASD | 0.04433 | 0.00764 | 254 | 5.7997 | < .001* |
|  | | TD | - | syllable | TD | 0.06615 | 0.00887 | 254 | 7.4617 | < .001* |
|  | |  | - | phoneme | TD | 0.08900 | 0.00887 | 254 | 10.0395 | < .001* |
| syllable | | ASD | - | syllable | TD | -0.00361 | 0.00855 | 378 | -0.4222 | 1.000 |
|  | |  | - | phoneme | ASD | 0.01996 | 0.00764 | 254 | 2.6120 | 0.143 |
|  | | TD | - | phoneme | TD | 0.02285 | 0.00887 | 254 | 2.5777 | 0.158 |
| phoneme | | ASD | - | phoneme | TD | -7.18e−4 | 0.00855 | 378 | -0.0841 | 1.000 |

**
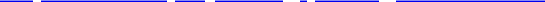
**

* = Significance based on False Discovery Rate (FDR), SE = Standard error, df = degrees of freedom

**・Transfer Entropy analyses.**

**Within Subjects Effects**

|  | **Sum of Squares** | **df** | **Mean Square** | **F** | **p** | **η²p** |
| --- | --- | --- | --- | --- | --- | --- |
| Rhythm | 0.48047 | 1.57 | 0.30518 | 496.35 | < .001 | 0.796 |
|  | 0.48047 | 1.57 | 0.30518 | 496.35 | < .001 | 0.796 |
| Rhythm ┯ Group | 0.00359 | 1.57 | 0.00228 | 3.71 | 0.036 | 0.028 |
|  | 0.00359 | 1.57 | 0.00228 | 3.71 | 0.036 | 0.028 |
| Residual | 0.12294 | 199.95 | 6.15e-4 |  |  |  |
|  | 0.12294 | 199.95 | 6.15e-4 |  |  |  |
| Hierarchy | 0.76497 | 1 | 0.76497 | 1777.07 | < .001 | 0.933 |
| Hierarchy ┯ Group | 0.00650 | 1 | 0.00650 | 15.11 | < .001 | 0.106 |
| Residual | 0.05467 | 127 | 4.30e-4 |  |  |  |
| Rhythm ┯ Hierarchy | 0.22988 | 1.29 | 0.17758 | 536.89 | < .001 | 0.809 |
| Rhythm ┯ Hierarchy ┯ Group | 0.00251 | 1.29 | 0.00194 | 5.86 | 0.010 | 0.044 |
| Residual | 0.05438 | 164.41 | 3.31e-4 |  |  |  |

* = Significance based on False Discovery Rate (FDR), SE = Standard error, df = degrees of freedom

**Post Hoc Comparisons - Rhythm**

| **Rhythm** |  | **Rhythm** | **Mean Difference** | **SE** | **df** | **t** | **p(FDR)** |
| --- | --- | --- | --- | --- | --- | --- | --- |
| Pro-Sy | - | Pro-Ph | 0.04980 | 0.00196 | 254 | 25.43 | < .001* |
|  | - | Sy-Ph | -0.00665 | 0.00196 | 254 | -3.40 | < .001* |
| Pro-Ph | - | Sy-Ph | -0.05645 | 0.00196 | 254 | -28.82 | < .001* |

* = Significance based on False Discovery Rate (FDR), SE = Standard error, df = degrees of freedom

**Post Hoc Comparisons - Hierarchy**

| **Hierarchy** | **Hierarchy** | **Mean Difference** | **SE** | **df** | **t** | **p** |
| --- | --- | --- | --- | --- | --- | --- |
| High->Low | Low->High | 0.0636 | 0.00151 | 127 | 42.2 | < .001 |

* = Significance based on False Discovery Rate (FDR), SE = Standard error, df = degrees of freedom

**Post Hoc Comparisons - Rhythm** ┯ **Hierarchy**

| **Rhythm** | **Hierarchy** |  | **Rhythm** | **Hierarchy** | **Mean Difference** | **SE** | **df** | **t** | **p(FDR)** |
| --- | --- | --- | --- | --- | --- | --- | --- | --- | --- |
| Pro-Sy | High->Low | - | Pro-Sy | Low->High | 0.09567 | 0.00213 | 338 | 44.92 | < .001* |
|  |  | - | Pro-Ph | High->Low | 0.05769 | 0.00235 | 442 | 24.53 | < .001* |
|  |  | - | Sy-Ph | High->Low | 0.03362 | 0.00235 | 442 | 14.29 | < .001* |
|  | Low->High | - | Pro-Ph | Low->High | 0.04191 | 0.00235 | 442 | 17.82 | < .001* |
|  |  | - | Sy-Ph | Low->High | -0.04692 | 0.00235 | 442 | -19.95 | < .001* |
| Pro-Ph | High->Low | - | Pro-Ph | Low->High | 0.07990 | 0.00213 | 338 | 37.52 | < .001* |
|  |  | - | Sy-Ph | High->Low | -0.02407 | 0.00235 | 442 | -10.23 | < .001* |
|  | Low->High | - | Sy-Ph | Low->High | -0.08883 | 0.00235 | 442 | 37.77 | < .001* |
| Sy-Ph | High->Low | - | Sy-Ph | Low->High | 0.01514 | 0.00213 | 338 | 7.11 | < .001* |

* = Significance based on False Discovery Rate (FDR), SE = Standard error, df = degrees of freedom

**Post Hoc Comparisons - Rhythm** ┯ **Group**

| **Rhythm** | **Group** |  | **Rhythm** | **Group** | **Mean Difference** | **SE** | **df** | **t** | **p(FDR)** |
| --- | --- | --- | --- | --- | --- | --- | --- | --- | --- |
| Pro-Sy | ASD | - | Pro-Sy | TD | -0.03897 | 0.00627 | 166 | -6.213 | < .001* |
|  |  | - | Pro-Ph | ASD | 0.04466 | 0.00256 | 254 | 17.464 | < .001* |
|  |  | - | Sy-Ph | ASD | -0.01047 | 0.00256 | 254 | -4.095 | < .001* |
|  | TD | - | Pro-Ph | TD | 0.05494 | 0.00297 | 254 | 18.519 | < .001* |
|  |  | - | Sy-Ph | TD | -0.00283 | 0.00297 | 254 | -0.952 | 0.342 |
| Pro-Ph | ASD | - | Pro-Ph | TD | -0.02870 | 0.00627 | 166 | -4.575 | < .001* |
|  |  | - | Sy-Ph | ASD | -0.05514 | 0.00256 | 254 | -21.559 | < .001* |
|  | TD | - | Sy-Ph | TD | -0.05776 | 0.00297 | 254 | -19.471 | < .001* |
| Sy-Ph | ASD | - | Sy-Ph | TD | -0.03132 | 0.00627 | 166 | -4.993 | < .001* |

* = Significance based on False Discovery Rate (FDR), SE = Standard error, df = degrees of freedom

**Post Hoc Comparisons - Hierarchy** ┯ **Group**

| **Hierarchy** | **Group** |  | **Hierarchy** | **Group** | **Mean Difference** | **SE** | **df** | **t** | **p(FDR)** |
| --- | --- | --- | --- | --- | --- | --- | --- | --- | --- |
| High->Low | ASD | - | High->Low | TD | -0.0389 | 0.00604 | 144 | -6.43 | < .001* |
|  |  | - | Low->High | ASD | 0.0577 | 0.00197 | 127 | 29.30 | < .001* |
|  | TD | - | Low->High | TD | 0.0694 | 0.00228 | 127 | 30.40 | < .001* |
| Low->High | ASD | - | Low->High | TD | -0.0271 | 0.00604 | 144 | -4.49 | < .001* |

* = Significance based on False Discovery Rate (FDR), SE = Standard error, df = degrees of freedom

**Post Hoc Comparisons - Rhythm** ┯ **Hierarchy** ┯ **Group**

| **Rhythm** | **Hierarchy** | **Group** |  | **Rhythm** | **Hierarchy** | **Group** | **Mean Difference** | **SE** | **df** | **t** | **p(FDR)** |
| --- | --- | --- | --- | --- | --- | --- | --- | --- | --- | --- | --- |
| Pro-Sy | High->Low | ASD | - | Pro-Sy | High->Low | TD | -0.0489 | 0.00662 | 205 | -7.382 | < .001* |
|  |  |  | - | Pro-Sy | Low->High | ASD | 0.08574 | 0.00278 | 338 | 30.827 | < .001* |
|  |  |  | - | Pro-Ph | High->Low | ASD | 0.05086 | 0.00307 | 442 | 16.559 | < .001* |
|  |  |  | - | Sy-Ph | High->Low | ASD | 0.02538 | 0.00307 | 442 | 8.262 | < .001* |
|  |  | TD | - | Pro-Sy | Low->High | TD | 0.1056 | 0.00323 | 338 | 32.734 | < .001* |
|  |  |  | - | Pro-Ph | High->Low | TD | 0.06451 | 0.00356 | 442 | 18.107 | < .001* |
|  |  |  | - | Sy-Ph | High->Low | TD | 0.04186 | 0.00356 | 442 | 11.75 | < .001* |
|  | Low->High | ASD | - | Pro-Sy | Low->High | TD | -0.02904 | 0.00662 | 205 | -4.384 | < .001* |
|  |  |  | - | Pro-Ph | Low->High | ASD | 0.03847 | 0.00307 | 442 | 12.524 | < .001* |
|  |  |  | - | Sy-Ph | Low->High | ASD | -0.04632 | 0.00307 | 442 | -15.082 | < .001* |
|  |  | TD | - | Pro-Ph | Low->High | TD | 0.04536 | 0.00356 | 442 | 12.733 | < .001* |
|  |  |  | - | Sy-Ph | Low->High | TD | -0.04751 | 0.00356 | 442 | -13.336 | < .001* |
| Pro-Ph | High->Low | ASD | - | Pro-Ph | High->Low | TD | -0.03525 | 0.00662 | 205 | -5.322 | < .001* |
|  |  |  | - | Pro-Ph | Low->High | ASD | 0.07334 | 0.00278 | 338 | 26.371 | < .001* |
|  |  |  | - | Sy-Ph | High->Low | ASD | -0.02548 | 0.00307 | 442 | -8.297 | < .001* |
|  |  | TD | - | Pro-Ph | Low->High | TD | 0.08645 | 0.00323 | 338 | 26.798 | < .001* |
|  |  |  | - | Sy-Ph | High->Low | TD | -0.02265 | 0.00356 | 442 | -6.358 | < .001* |
|  | Low->High | ASD | - | Pro-Ph | Low->High | TD | -0.02214 | 0.00662 | 205 | -3.343 | < .001* |
|  |  |  | - | Sy-Ph | Low->High | ASD | -0.08479 | 0.00307 | 442 | -27.606 | < .001* |
|  |  | TD | - | Sy-Ph | Low->High | TD | -0.09287 | 0.00356 | 442 | -26.068 | < .001* |
| Sy-Ph | High->Low | ASD | - | Sy-Ph | High->Low | TD | -0.03242 | 0.00662 | 205 | -4.894 | < .001* |
|  |  |  | - | Sy-Ph | Low->High | ASD | 0.01404 | 0.00278 | 338 | 5.047 | < .001* |
|  |  | TD | - | Sy-Ph | Low->High | TD | 0.01623 | 0.00323 | 338 | 5.032 | < .001* |
|  | Low->High | ASD | - | Sy-Ph | Low->High | TD | -0.03023 | 0.00662 | 205 | -4.563 | < .001* |

* = Significance based on False Discovery Rate (FDR), SE = Standard error, df = degrees of freedom
