# Supplementary material for: Non-autistic persons modulate their speech rhythm while talking to autistic individuals
Source: PLoS One. 2023 Sep 28;18(9):e0285591. doi: 10.1371/journal.pone.0285591 (PMC10538692; doi:10.1371/journal.pone.0285591)
Supplement: S3 Appendix — (DOCX) [file pone.0285591.s003.docx]

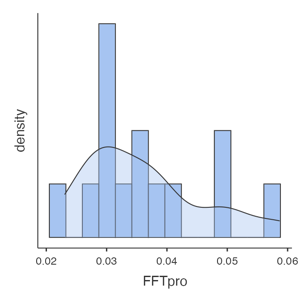

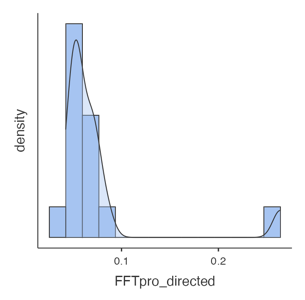
**・Normality test**

**FFT prosody_directed**

**FFT prosody**

|  | FFT prosody | FFT  pro_directed |
| --- | --- | --- |
| N | 14 | 14 |
| Shapiro-Wilk W | 0.915 | 0.488 |
| Shapiro-Wilk (p) | 0.185 | < .001 |

|  | Pro>Sy | Pro>Ph | Sy->Ph | Pro->Sy_directed | Pro->Ph_directed | Sy->Ph_directed |
| --- | --- | --- | --- | --- | --- | --- |
| N | 14 | 14 | 14 | 14 | 14 | 14 |
| Shapiro-Wilk W | 0.838 | 0.797 | 0.841 | 0.949 | 0.962 | 0.956 |
| Shapiro-Wilk (p) | 0.015 | 0.005 | 0.017 | 0.544 | 0.748 | 0.664 |


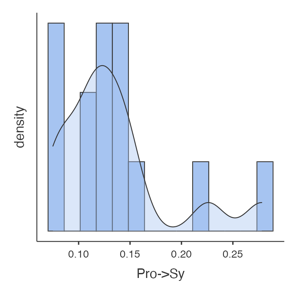

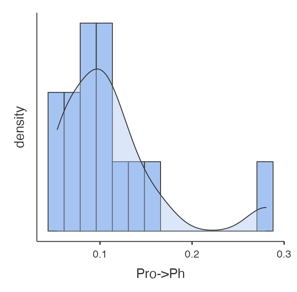

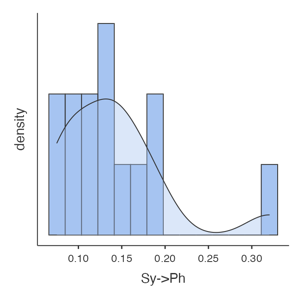

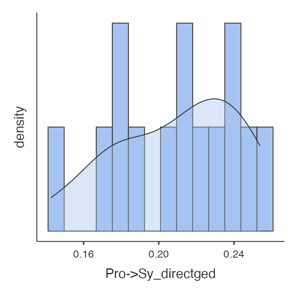

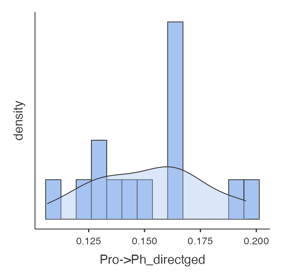

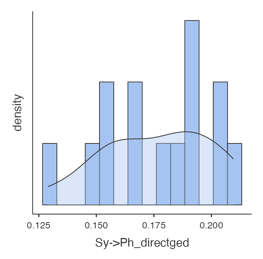


**Prosody->Syllable**

**Prosody->Phoneme**

**Syllable->Phoneme**

**Prosody->Syllable_directed**

**Prosody->Phoneme_directed**

**Syllable->Phoneme_directed**

|  | Sy-  >Pro | Ph-  >Sy | Ph-  >Pro | Sy-  >Pro_directed | Ph-  >Pro_directed | Ph->Sy_directed |
| --- | --- | --- | --- | --- | --- | --- |
| N | 14 | 14 | 14 | 14 | 14 | 14 |
| Shapiro-Wilk W | 0.855 | 0.888 | 0.841 | 0.929 | 0.952 | 0.945 |
| Shapiro-Wilk (p) | 0.026 | 0.077 | 0.017 | 0.298 | 0.590 | 0.483 |


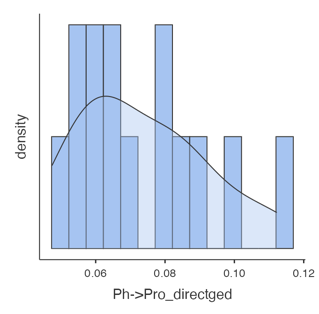

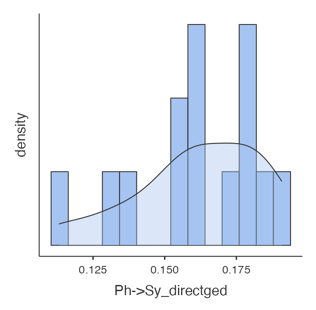

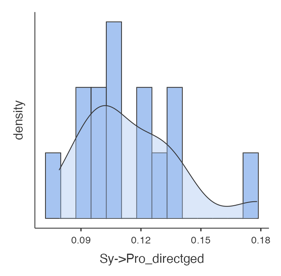

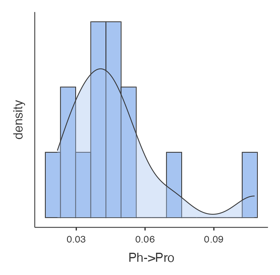

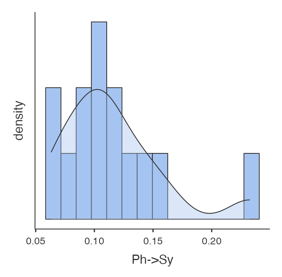

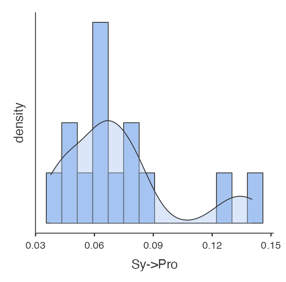


**Syllable->Prosody**

**Phoneme->Syllable**

**Phoneme->Prosody**

**Prosody->Syllable_directed**

**Prosody->Phoneme_directed**

**Syllable->Phoneme_directed**

**・Correlation test**

**・FFT**

|  |  | FFT prosody |
| --- | --- | --- |
| FFT prosody_directed | Spearman correlation coefficient | 0.490 |
|  | p value | 0.039 |

* Alternative hypothesis：Positive correlation


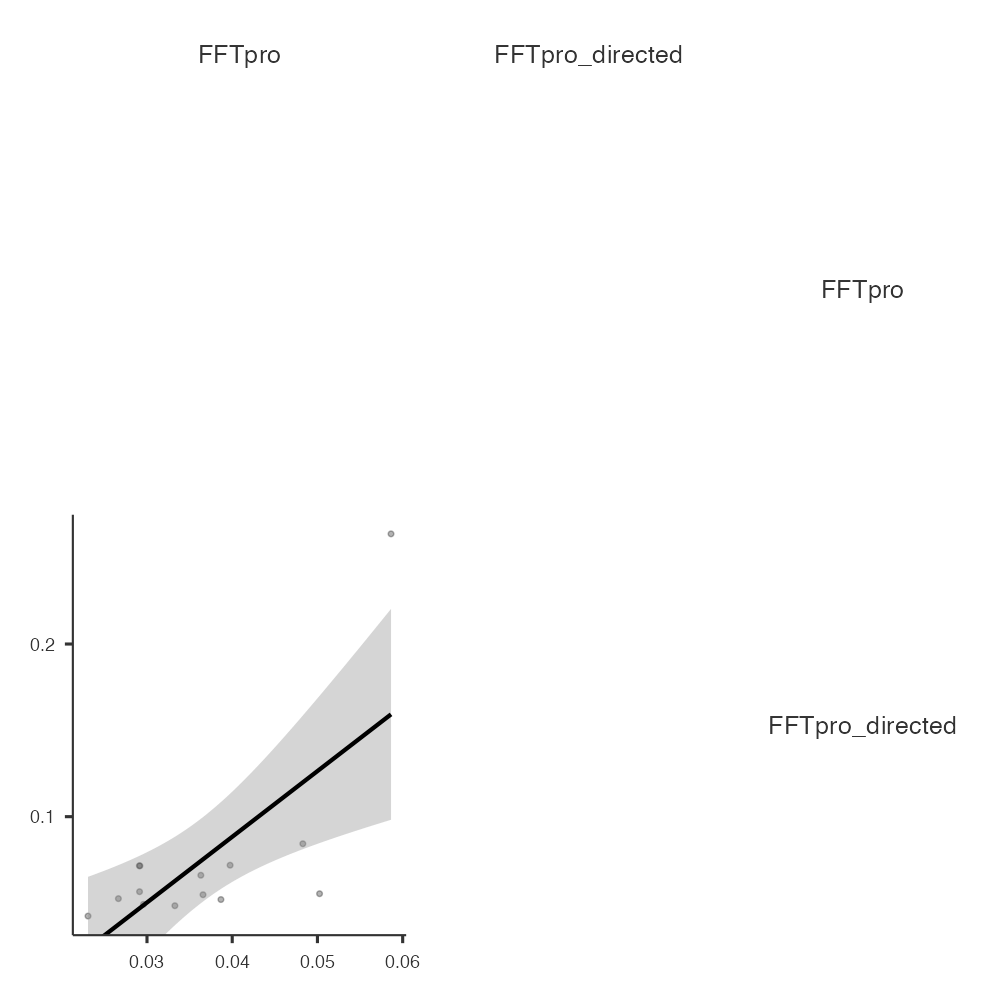


**Prosody directed**

**Prosody**

**・Transfer entropy analysis**

|  |  | Prosody->Syllable |
| --- | --- | --- |
| Prosody->Syllable_directed | Spearman correlation coefficient | 0.345 |
|  | p value | 0.114 |

* Alternative hypothesis：Positive correlation


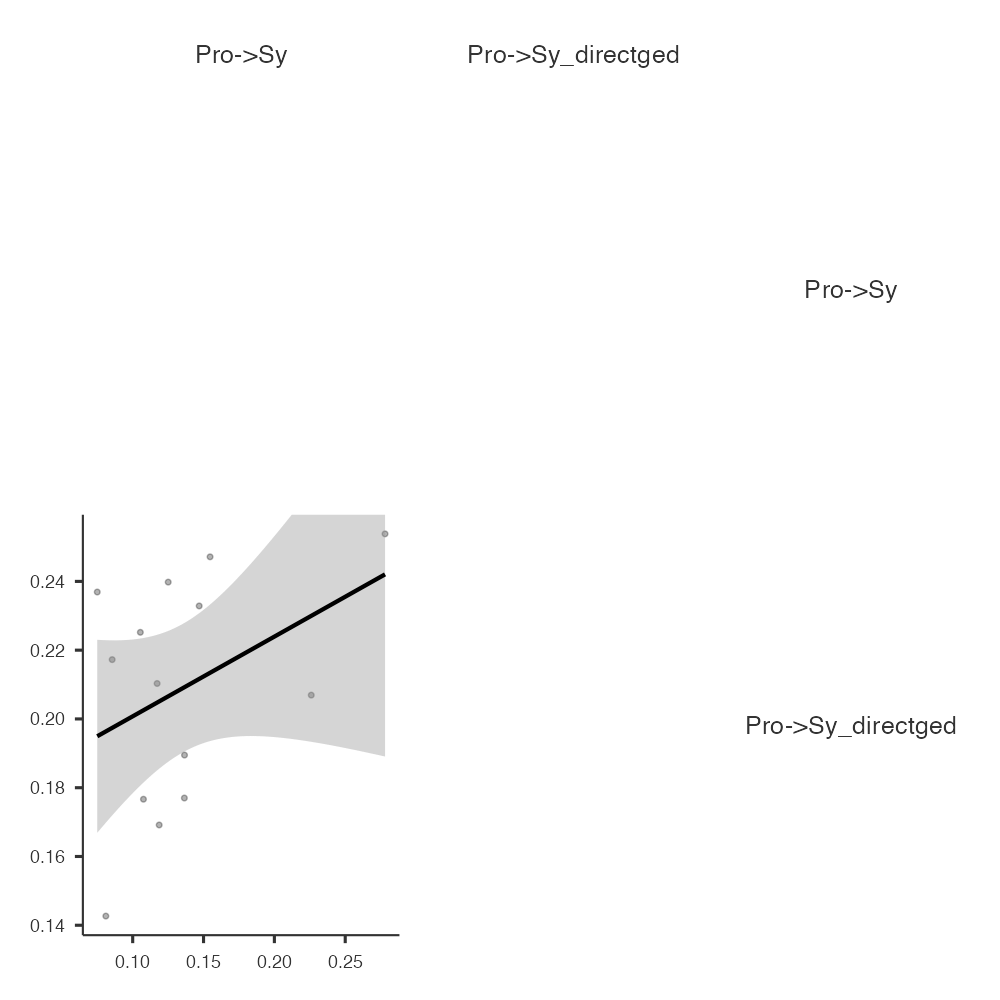


**Prosody->Syllable_directed**

**Prosody->Syllable**

|  |  | Prosody->Phoneme |
| --- | --- | --- |
| Prosody->Phoneme_directed | Spearman correlation coefficient | 0.257 |
|  | p value | 0.187 |

* Alternative hypothesis：Positive correlation


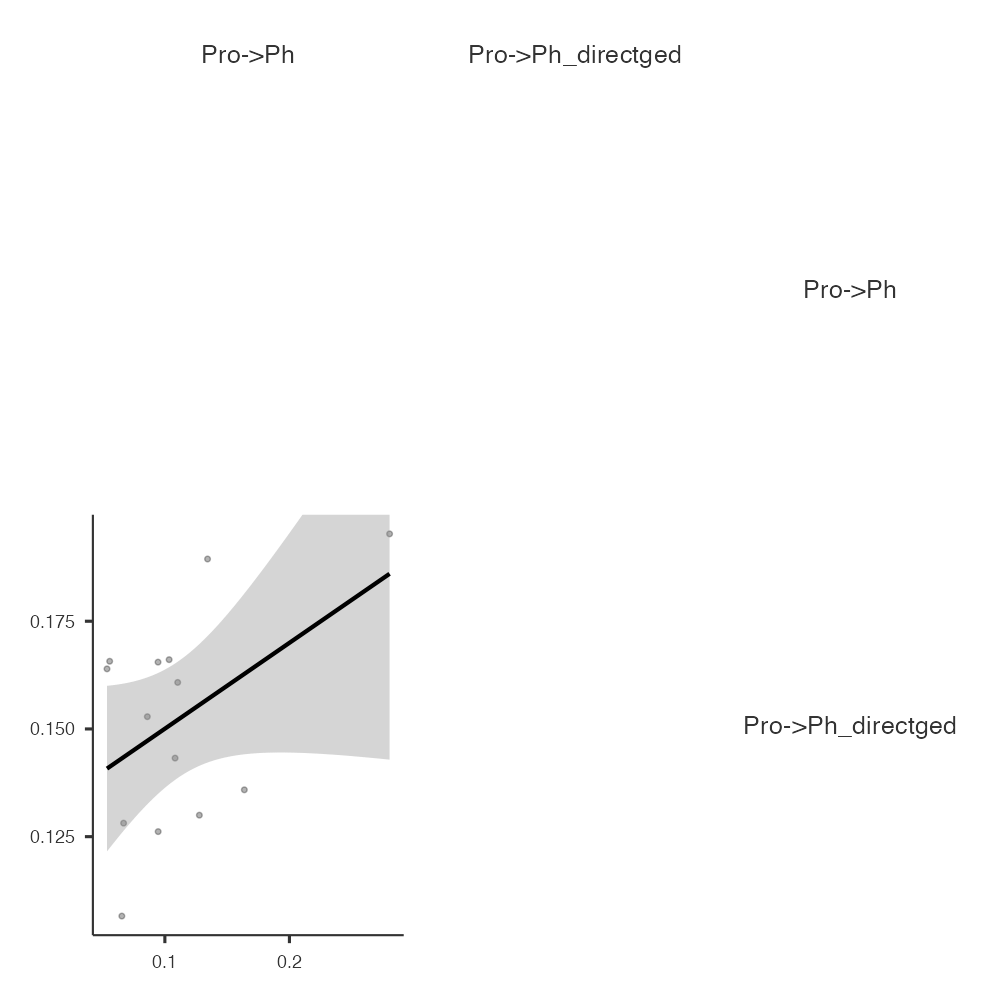


**Prosody->Phoneme_directed**

**Prosody->Phoneme**

|  |  | Syllable->Phoneme |
| --- | --- | --- |
| Syllable->Phoneme_directed | Spearman correlation coefficient | 0.086 |
|  | p value | 0.387 |

* Alternative hypothesis：Positive correlation
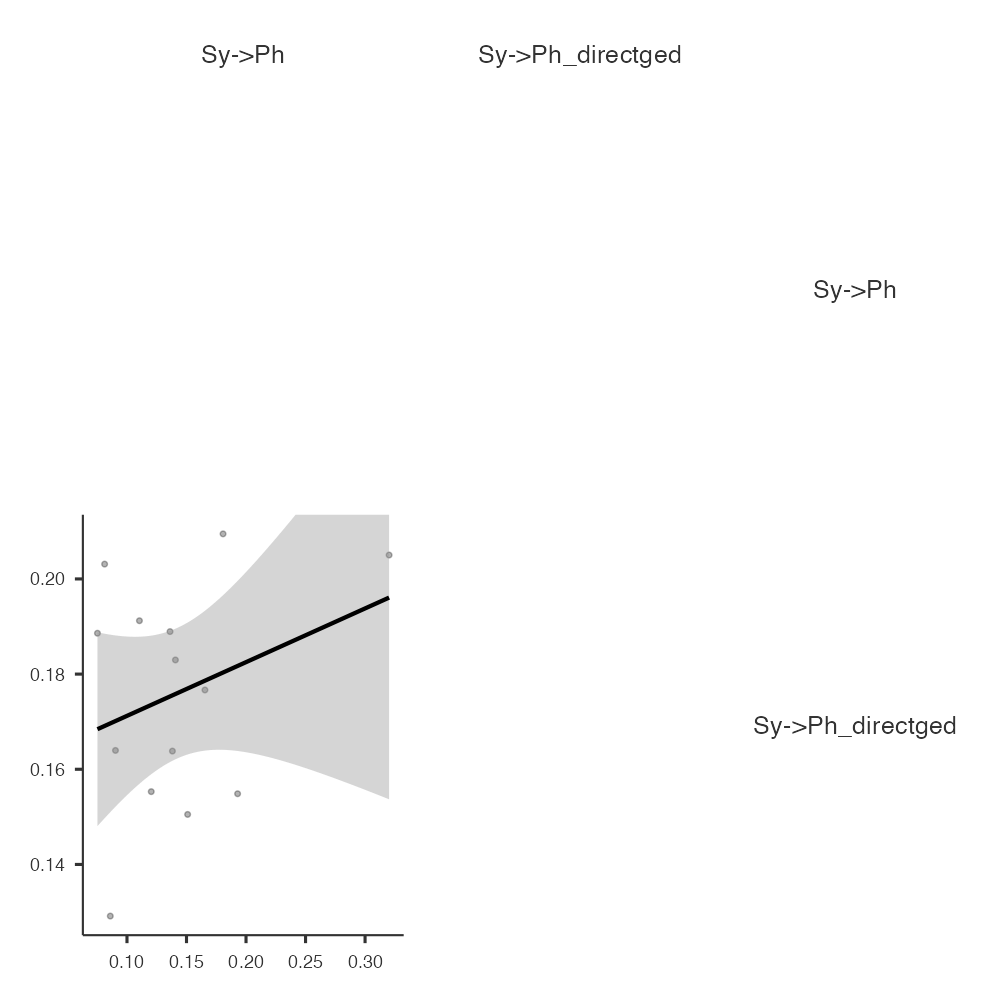


**Syllable->Phoneme_directed**

**Syllable->Phoneme**

|  |  | Syllable->Prosody |
| --- | --- | --- |
| Sy->Pro_directed | Spearman correlation coefficient | 0.393 |
|  | p value | 0.083 |

* Alternative hypothesis：Positive correlation

|  |  | Phoneme>Prosody |
| --- | --- | --- |
| Phoneme->Prosody_directed | Spearman correlation coefficient | 0.486 |
|  | p value | 0.040 |


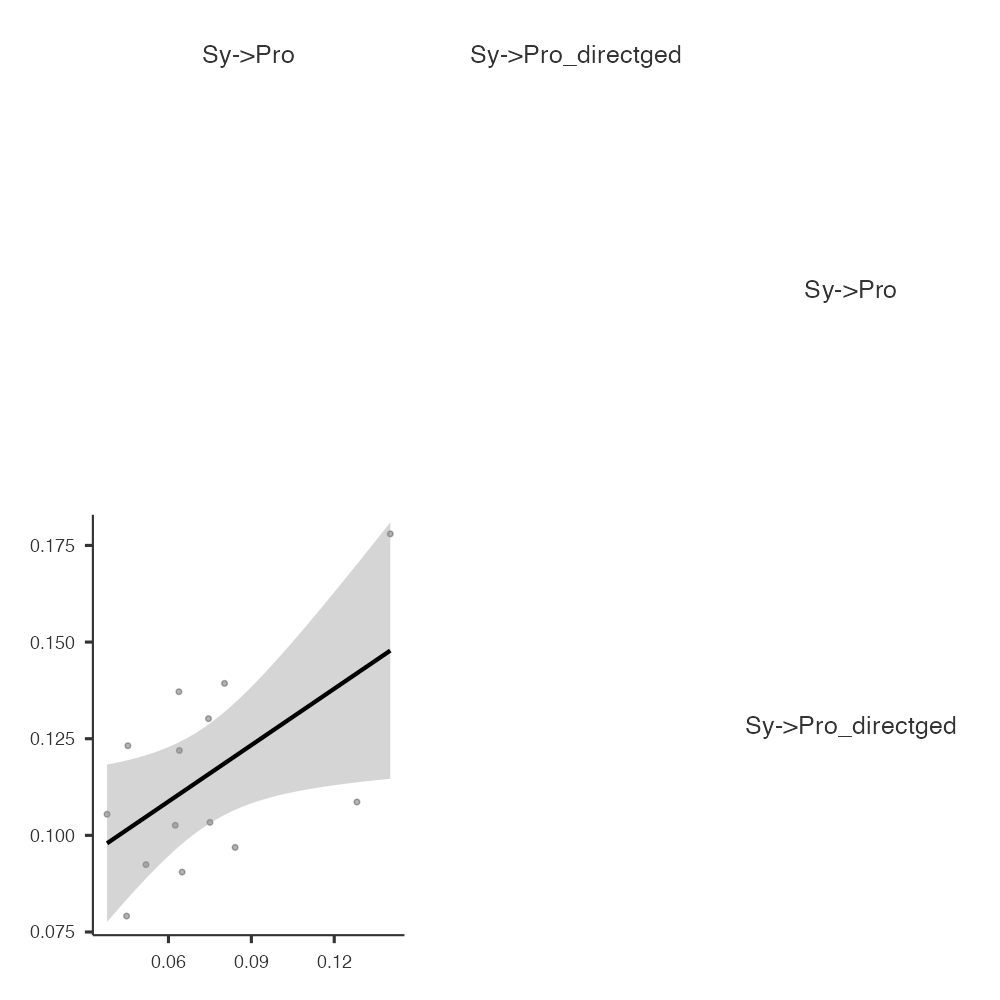


**Syllable->Phoneme_directed**


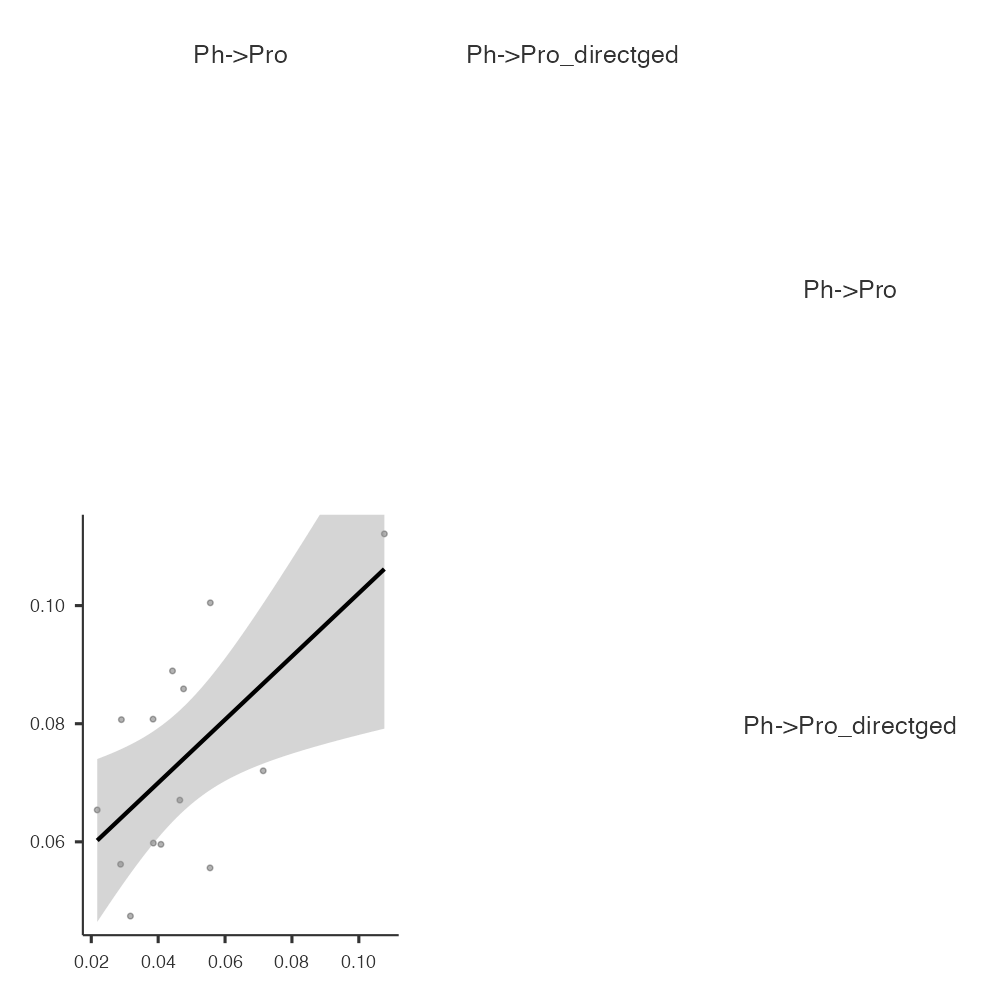


**Phoneme->Prosody_directed**

**Phoneme->Prosody**

**Syllable->Phoneme**

|  |  | Phoneme->Syllable |
| --- | --- | --- |
| Phoneme->Syllable_directed | Spearman correlation coefficient | 0.301 |
|  | p value | 0.148 |


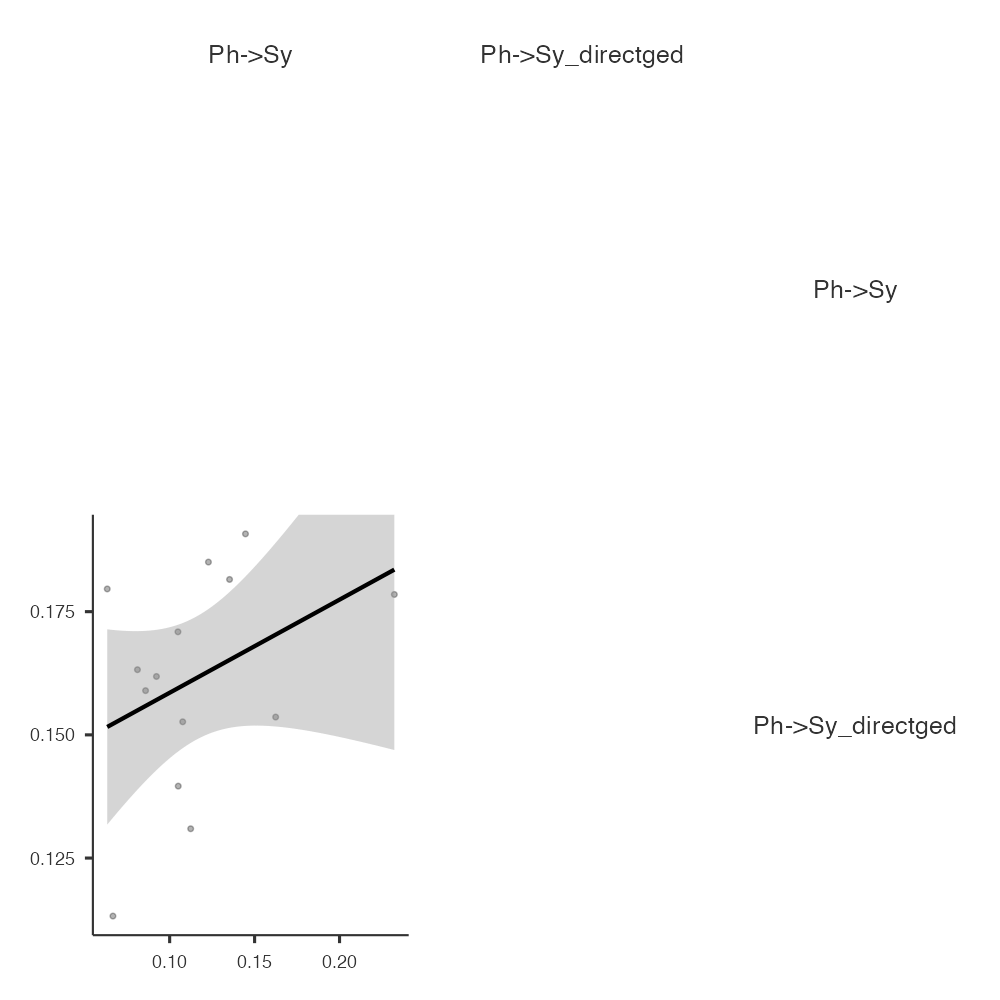


**Phoneme->Syllable**

**Phoneme->Syllable_directed**
