# Supplementary figures and images for: Non-autistic persons modulate their speech rhythm while talking to autistic individuals
Source: PLoS One. 2023 Sep 28;18(9):e0285591. doi: 10.1371/journal.pone.0285591 (PMC10538692; doi:10.1371/journal.pone.0285591)

**Spectrogram and scalogram in a speech signal.**

**
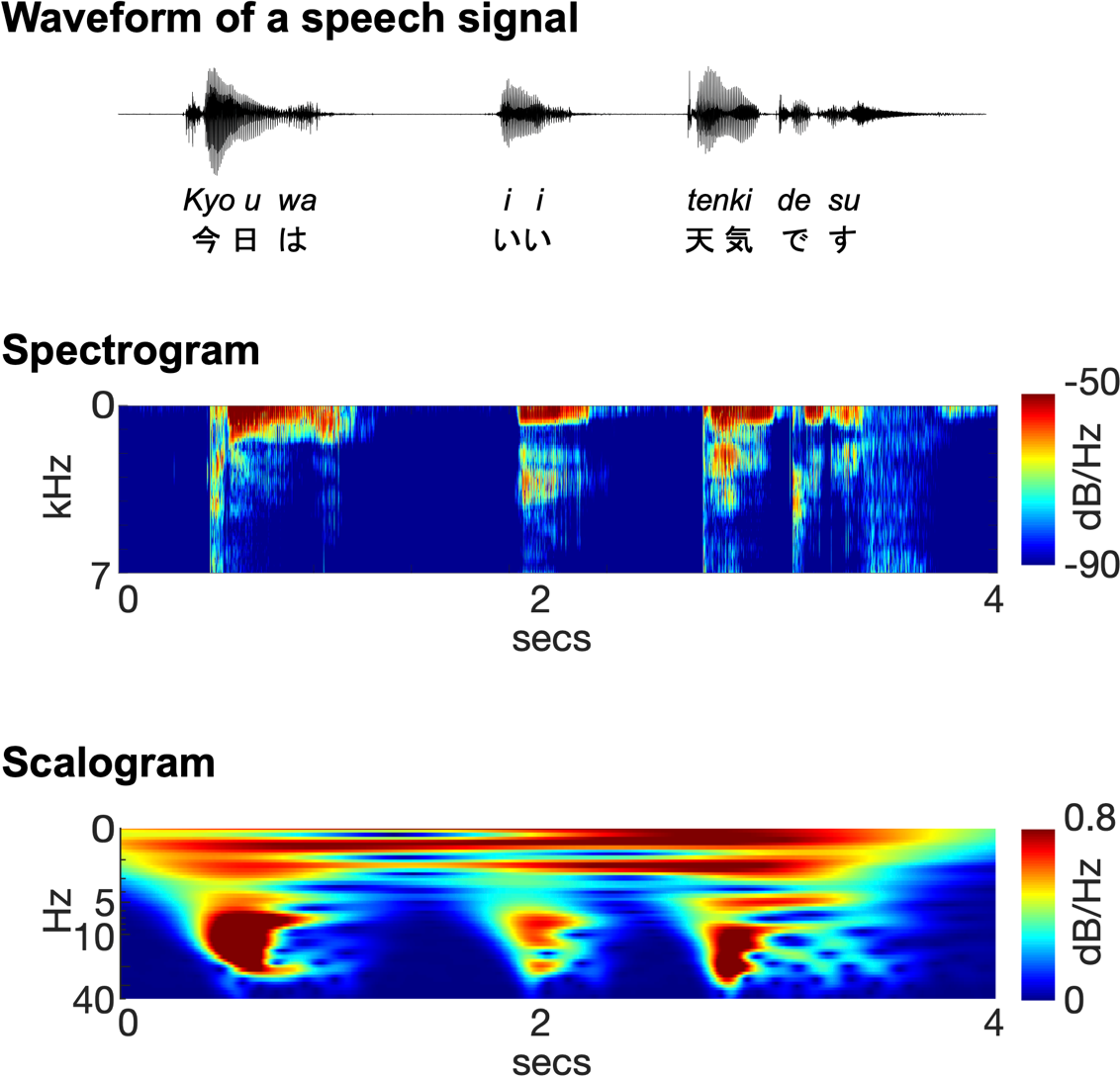
**

Supplement: S4 Appendix — (DOC) [file pone.0285591.s004.doc]
