# Supplementary material for: Non-autistic persons modulate their speech rhythm while talking to autistic individuals
Source: PLoS One. 2023 Sep 28;18(9):e0285591. doi: 10.1371/journal.pone.0285591 (PMC10538692; doi:10.1371/journal.pone.0285591)
Supplement: Attachment — Submitted filename: Response_to_Reviewer.docx [file pone.0285591.s005.docx]

**RESPONSE TO EDITORS:**

We are extremely grateful for the Editors’ insightful comments on our manuscript. We have studied the comments carefully and have made the necessary corrections in our paper. Our responses to the comments are as follows.

**Comment 1:**

Please ensure that your manuscript meets PLOS ONE's style requirements, including those for file naming. The PLOS ONE style templates can be found at

https://journals.plos.org/plosone/s/file?id=wjVg/PLOSOne_formatting_sample_main_body.pdf and

https://journals.plos.org/plosone/s/file?id=ba62/PLOSOne_formatting_sample_title_authors_affiliations.pdf

**Response:** Thank you for the comment. We have ensured that the manuscript meets PLOS ONE's style requirements. We have also asked a language editing service to check the writing and ensure that it meets PLOS ONE's style.

**Comment 2:**

Please change "female” or "male" to "woman” or "man" as appropriate, when used as a noun (see for instance https://apastyle.apa.org/style-grammar-guidelines/bias-free-language/gender).

**Response:** Thank you for the comment. We have changed the word accordingly.

**Comment 3:**

We note that the grant information you provided in the ‘Funding Information’ and ‘Financial Disclosure’ sections do not match. When you resubmit, please ensure that you provide the correct grant numbers for the awards you received for your study in the ‘Funding Information’ section.

We note that you have provided funding information that is not currently declared in your Funding Statement. However, funding information should not appear in the Acknowledgments section or other areas of your manuscript. We will only publish funding information present in the Funding Statement section of the online submission form. Please remove any funding-related text from the manuscript and let us know how you would like to update your Funding Statement.

**Response:** Thank you very much for the comment. We have updated and confirmed them through the online system. We have also removed any funding-related text from the manuscript. All the authors declare no competing financial interests. moreover, we have amended statements in the cover letter as follows:

‘This research was supported by JST CREST 'Cognitive Feelings' (Grant Number: JPMJCR21P4), JSPS KAKENHI (Grant Number 22KK0157, 22H05210, 21H05063, 21H05053), JST Moonshot Goal 9(JPMJMS2296), Institute for AI and Beyond, the University of Tokyo, and World Premier International Research Centre Initiative (WPI), MEXT, Japan. The funders had no role in study design, data collection and analysis, decision to publish, or preparation of the manuscript.’

**Comment 4:**

We note that you have indicated that data from this study are available upon request. PLOS only allows data to be available upon request if there are legal or ethical restrictions on sharing data publicly. For more information on unacceptable data access restrictions, please see http://journals.plos.org/plosone/s/data-availability#loc-unacceptable-data-access-restrictions.

In your revised cover letter, please address the following prompts:

a) If there are ethical or legal restrictions on sharing a de-identified data set, please explain them in detail (e.g., data contain potentially sensitive information, data are owned by a third-party organization, etc.) and who has imposed them (e.g., an ethics committee). Please also provide contact information for a data access committee, ethics committee, or other institutional body to which data requests may be sent.

b) If there are no restrictions, please upload the minimal anonymized data set necessary to replicate your study findings as either Supporting Information files or to a stable, public repository and provide us with the relevant URLs, DOIs, or accession numbers. For a list of acceptable repositories, please see http://journals.plos.org/plosone/s/data-availability#loc-recommended-repositories.

We will update your Data Availability statement on your behalf to reflect the information you provide.

**Response:** We thank the reviewer for the comments. All the minimal anonymized data set are available in supporting information (S1, S2, and S3 Appendix). We have stated that the data set can be found under the section of Data Availability.

**Comment 5:**

Additional Editor Comments:

This research is very interesting, and the idea is unique. However, due to some problems such as the number of samples, this study cannot be accepted at this time. The sample size should be stated in the abstract so as not to mislead the reader.

**Response:** We thank the reviewer for the important comments. We stated the sample size in the abstract section so as not to mislead potential readers as follows.

‘Eight adults diagnosed with AUT (all men; age range, 24–44 years) and eight age-matched non-autistic NT adults (three women, five men; age range, 23–45 years) participated in this study. Six NT and eight AUT respondents were asked by one of the two NT questioners (all men) to share their recent experiences on 12 topics. We included 87 samples of AUT-directed speech (from an NT questioner to an AUT respondent), 72 of NT-directed speech (from an NT questioner to an NT respondent), 74 of AUT speech (from an AUT respondent to an NT questioner), and 55 of NT speech (from an NT respondent to an NT questioner).’

**RESPONSE TO REVIEWER 1:**

I wish to express our strong appreciation to the Reviewer for the insightful comments on our manuscript. We have studied the Reviewer’s comments very carefully and have made necessary corrections. We feel the comments have helped us significantly improve the manuscript.

**Comment 1:**

Despite the authors noted that frequency variations are greater for AUT, only eight individuals with ASD participated in this study. Due to the small sample size of participants with ASD, there is little diversity in AUT utterances, and it would be difficult to show exactly how NT subjects adapt to AUT utterances.

**Response:** We thank the reviewer for the pertinent comments. We agree with the limitation of sample size. We clearly stated the sample size in the abstract section as to not mislead potential readers. Further, we added a correlation analysis to check not only general differences between AUT-directed speech and NT-directed speech, but also whether each NT individual changes their way of speaking depending on the speech characteristics of the interlocutor. The results suggested that the prosodic powers in NT questioners were positively correlated with those in NT and AUT respondents. This implies that an NT individual may change their way of speaking depending on the prosodic characteristics of the interlocutor. We included it in the Methods section, and as a limitation in the Discussion section as follows:

Abstract section

“Eight adults diagnosed with AUT (all men; age range, 24–44 years) and eight age-matched non-autistic NT adults (three women, five men; age range, 23–45 years) participated in this study. Six NT and eight AUT respondents were asked by one of the two NT questioners (all men) to share their recent experiences on 12 topics. We included 87 samples of AUT-directed speech (from an NT questioner to an AUT respondent), 72 of NT-directed speech (from an NT questioner to an NT respondent), 74 of AUT speech (from an AUT respondent to an NT questioner), and 55 of NT speech (from an NT respondent to an NT questioner).”

Methods section

‘Further, we averaged the frequency power of prosody and transfer entropy for each participant. We then performed a Shapiro–Wilk test for normality separately for the datasets containing frequency power of prosody and transfer entropy. Then, to understand whether each NT questioner changes their way of speaking depending on the speech characteristics of respondents, we performed a one-tailed Pearson’s or Spearman’s correlation test for each frequency power and transfer entropy between NT questioners and all of NT and AUT respondents, based on an alternative hypothesis of positive correlation. We selected p-values of <0.05 as the threshold for statistical significance and used a false discovery rate method for post-hoc analysis and multiple testing of significant effects.’

Discussion section

‘Another limitation is that although this study used a sufficient sample for the statistical analysis methods (Table 1), and all samples were sufficiently gathered from all speakers in terms of numbers and durations, this study used a small sample size of individuals. Particularly, each respondent was asked by only one of the two NT questioners. Further, this study could not control for sex (gender) differences between groups. This suggests that as well as AUT-specific characteristics, participant-specific characteristics may have influenced our findings. Nevertheless, given the small sample size in this study, we also conducted correlation analysis to examine whether each individual NT questioner changes their way of speaking depending on the speech characteristics of respondents. The findings indicated the prosodic powers in NT questioners were positively correlated with those in NT and AUT respondents. Hence, this study suggested the possibility that an NT individual may change their way of speaking depending on the prosodic characteristics of interlocutor.’

**Comment 2:**

According to the previous research cited by the authors, prosody, syllable, and phoneme indicators in the AM hierarchy were analyzed using samples of child directed speech in English. Therefore, it is unclear whether this frequency index can be applied to the analysis of speech to share recent experiences between adults (with ASD) in Japanese.

**Response:** Thank you for the important comment. The previous research cited in this paper analysed both adult-directed speech and infant-directed speech (Leong et al., 2017). They then detected a similar frequency index for prosody, syllable, and phoneme indicators in the AM hierarchy. In addition, the model that we used in this study differs from that used by Leong et al. (2017). She used a model called S-AMPH and this study used a PAD model. In a previous study, we have already confirmed that the frequency index between S-AMPH and PAD is consistent in both adults and children (Daikoku and Goswami, 2022). Further, in this study we first checked the boundary as a qualitative analysis using scalogram. Then, based on the qualitative understanding, we decided the frequency index (prosody: 1‒4 Hz, syllable: 4‒12 Hz, phoneme: 12‒40 Hz). Therefore, with evidence from previous studies (Leong et al., 2017; Daikoku and Goswami, 2022) and the confirmation based on scalogram in this study, this frequency index can be applied to the analysis of adult-directed speech. We have described this in the Introduction and Methods sections as follows:

Introduction section

‘Evidence has shown that AM hierarchy of speech signals, including prosody, syllable, and phoneme, can be detected in the temporal structure of speech waveforms such as delta (<4 Hz), theta/alpha (4–12 Hz), and beta/low gamma (12–40 Hz), respectively, regardless of speech types [13,18].’

Methods section

‘Next, based on the qualitative understanding using scalogram, we decided the frequency index in AM envelopes (prosody: 1‒4 Hz, syllable: 4‒12 Hz, phoneme: 12‒40 Hz), and quantitatively analysed two types of speech characteristics using three AM envelopes. Previous research analysed both adult-directed speech and infant-directed speech [12] and detected a similar frequency index for prosody, syllable, and phoneme in the AM hierarchy. Thus, from the qualitative analyses based on scalogram in this study, we confirmed these frequency indices can be applied to the analysis of adult-directed speech.’

Leong, V., Kalashnikova, M., Burnham, D., & Goswami, U. (2017). The temporal modulation structure of infant-directed speech. Open Mind, 1(2), 78-90.

Daikoku, T., & Goswami, U. (2022) Hierarchical amplitude modulation structures and rhythm patterns: Comparing Western musical genres, song, and nature sounds to Babytalk. PloS one. 3033;17(10), e0275631

**Comment 3:**

It would be helpful for reader to understand this research if the number of participants, gender and age of the participants in this study are written in the abstract.

**Response:** We thank the reviewer for the comments. We stated them in the Abstract section as follows:

‘Eight adults diagnosed with AUT (all men; age range, 24–44 years) and eight age-matched non-autistic NT adults (three women, five men; age range, 23–45 years) participated in this study. Six NT and eight AUT respondents were asked by one of the two NT questioners (all men) to share their recent experiences on 12 topics. We included 87 samples of AUT-directed speech (from an NT questioner to an AUT respondent), 72 of NT-directed speech (from an NT questioner to an NT respondent), 74 of AUT speech (from an AUT respondent to an NT questioner), and 55 of NT speech (from an NT respondent to an NT questioner).’

**Comment 4:**

Introduction

The authors mentioned that ‘AUT is a developmental disorder characterized by differences in social skills and communication’, but the definition of Autism Spectrum Disorder also includes Restricted, repetitive patterns of behavior.

**Response:** Thank you for the comment. As indicated, we have revised this sentence in the Introduction section as follows.

‘AUT is a developmental disorder characterised by differences in social skills, communication, and repetitive patterns of behaviour’

**Comment 5:**

Introduction

Two papers are cited on ‘associations between prosodic performance and better communication between AUT and neurotypical (NT) individuals’, although the authors describe it as ‘a paper’.

**Response:** Thank you very much for letting me know the typo. We modified it in the Introduction section as follows.

‘Previous studies have reported important associations between prosodic performance and better communication between AUT and neurotypical (NT) individuals [10,11].’

**Comment 6:**

Introduction

The basis for hypothesizing weaker interactions between different phonological hierarchy levels in AUT speech than in NT speech is unclear.

**Response:** We thank the reviewer for the pertinent comments. As indicated, we specified it more clearly in the Introduction section as follows:

‘Previous evidence suggests that the interaction between different phonological rhythms (e.g., prosody vs. syllable) reflects the intelligibility of speech rhythm patterns [20,21], implying that the interaction of prosodic (delta) and syllabic (theta) rhythms is key to understanding the speech characteristics [25]. Further, it has been shown that the weak interaction between prosodic and syllable rhythms can be detected when the power of prosodic rhythm is weak [12,18]. From these findings, we hypothesised that AUT speech that often exhibits weak prosody also shows a weak dynamical interaction between different phonological rhythms.’

**Comment 7:**

Fig.2. It would be better to add Japanese syllables that match the sound waveform.

**Response:** Thank you for the comment. Although it is difficult to insert all the Japanese syllables corresponding to these waveforms on the graphs, we have done so in figure 2 as shown.

**RESPONSE TO REVIEWER 2:**

We are extremely grateful for the Reviewers’ insightful comments on our manuscript. We have studied the Reviewers’ comments carefully and have made the necessary corrections in our paper. We believe that the comments have helped us significantly improve and refine the manuscript.

**Comment 1:**

The authors presented actual demographics on the questioners and respondents only at the end of the manuscript. This is possibly due to the text format, but on P6, L2 they state ‘all the questioners’, suggesting a fair number of NT questioners directing speech to NT and AUT individuals. However, it took until P13 before they mention the exact number of questioners: two (2). Not doing this earlier and just focusing on sample numbers is misleading the reader, given the generalized statements made overall about speech of NT individuals directed to AUT persons. Furthermore, the AUT group (all males) and the NT group (5 males, 3 females) are composed of persons with inherently different voice characteristics (e.g. gender differences in the use of prosody), and it is unknown whether all samples were gathered equally from all speakers, so there is ample possibility that results were biased. Furthermore, there are various descriptions of the duration of the samples (see points 18 and 19 below), raising concerns about sample selection and the overall replicability of the analyses. The authors should clarify these issues or prove otherwise, and until then in my opinion the manuscript is unsuitable for publication.

**Response:** We thank the reviewer for the pertinent comments. As suggested, we have stated the sample size in the abstract section so as not to mislead potential readers. Further, we have specified information regarding gender in the first paragraph of the Results section so that potential readers are informed before reading the Results and Discussion sections. Then, we have clearly stated the limitation of this study in the Discussion section as mentioned below. Further, given the concern about sample size, we also conducted a correlation analysis to not only check for general difference between AUT-directed speech and NT-directed speech, but also to check whether each NT individual changes their way of speaking depending on the speech characteristics of interlocutor. The results suggested that the prosodic powers in NT questioners were positively correlated with those in NT and AUT respondents. This implies that an NT individual may change their way of speaking depending on the prosodic characteristics of interlocutor. We have also included this as a limitation in the Discussion section as follows. Finally, we have responded to all comments raised by the reviewer. Our changes are reflected in the text as follows:

Abstract section

‘Eight adults diagnosed with AUT (all men; age range, 24–44 years) and eight age-matched non-autistic NT adults (three women, five men; age range, 23–45 years) participated in this study.’

Result section

‘Eight adults diagnosed with AUT (all men; age range, 24–44 years) and eight age-matched non-autistic adults (three women, five men; age range, 23–45 years) participated in this study. Six NT and eight AUT respondents were asked by one of two NT questioners (all men) to share their recent experiences on 12 topics. We included 87 samples of AUT-directed speech (from an NT questioner to an AUT respondent), 72 samples of NT-directed speech (from an NT questioner to an NT respondent), 74 samples of AUT speech (from an AUT respondent to an NT questioner), and 55 samples of NT speech (from an NT respondent to an NT questioner).’

Discussion section

‘Another limitation is that although this study used a sufficient sample for the statistical analysis methods (Table 1), and all samples were sufficiently gathered from all speakers in terms of numbers and durations, this study used a small sample size of individuals. Particularly, each respondent was asked by only one of the two NT questioners. Further, this study could not control for sex (gender) differences between groups. This suggests that as well as AUT-specific characteristics, participant-specific characteristics may have influenced our findings. Nevertheless, given the small sample size in this study, we also conducted correlation analysis to examine whether each individual NT questioner changes their way of speaking depending on the speech characteristics of respondents. The findings indicated the prosodic powers in NT questioners were positively correlated with those in NT and AUT respondents. Hence, this study suggested the possibility that an NT individual may change their way of speaking depending on the prosodic characteristics of interlocutor.’

**Comment 2:**

Abstract, unnecessary spaces (L1, L4, L9) and period (L4, before “NT”).

**Response:** Thank you for the comment. We modified the unnecessary spaces throughout the manuscript.

**Comment 3:**

Acronym AUT is used liberally meaning “autistic” (adjective) or “autistic persons” (adj + noun) as in the last sentence of the abstract (“speech rhythms in AUT”). Please fix this in a consistent way (e.g, “speech rhythms produced by AUT persons”, or “rhythms in AUT speech”, or something like that).

**Response:** Thank you very much for the important comment. As indicated, we have revised it in the Abstract section as follows.

‘This suggests that NT individuals spontaneously imitate speech rhythms of the NT and AUT interlocutor.’

**Comment 4:**

Introduction, Characteristics of speech in ASD. The explanation in between lines 6-12 is not very smooth. L9 “not simple but peculiar in various ways” is not clear; frequency variations (e.g., pitch escalation / melodic variability) are related to prosody. L6 and L12 seem similar.

**Response:** We thank the reviewer for the pertinent comments. As indicated, we have revised the sentence in the section of ‘Characteristics of speech in autism spectrum disorder’ as follows.

‘Speech prosody, characterised by intonation, accentuation, and stress, is often weak and monotonous in AUT individuals (hereafter, ‘AUT speech’) [6] and has been typically utilised as a diagnostic marker of AUT [9].’

**Comment 5:**

P4, Phonological hierarchy in speech rhythm, first paragraph. Including a description in terms of delta, theta/alpha, beta/gamma rhythms would be informative, not just at the end of the paper in data analysis.

**Response:** Thank you for the comment. As suggested, we have described the information also in terms of delta, theta/alpha, beta/gamma rhythms in the section of ‘Phonological hierarchy in speech rhythm’ as follows.

‘Evidence has shown that AM hierarchy of speech signals, including prosody, syllable, and phoneme, can be detected in the temporal structure of speech waveforms such as delta (<4 Hz), theta/alpha (4–12 Hz), and beta/low gamma (12–40 Hz), respectively, regardless of speech types [13,18].’

**Comment 6:**

P4, Phonological hierarchy in speech rhythm, second paragraph. Fix the font.

**Response:** Thank you for the comment. We fixed the font.

**Comment 7:**

P4, Phonological hierarchy in speech rhythm, second paragraph, L7: the fact that “tenki” has 3 syllables is confusing to readers unfamiliar with Japanese, please explain.

**Response:** Thank you for the comment. As suggested, we explained it in the Introduction section as follows.

‘a parent node, such as the word ‘tenki’ at the prosodic level, would have three daughter nodes at the syllabic level, comprising the three syllables or moras (i.e., ‘te’, ‘n’, and ‘ki’).’

**Comment 8:**

Fig 1. Caption, L3, “Scalograms depicting ...” > “depict”

**Response:** Thank you for the indication. As indicated, we have revised it as follows.

‘Scalograms depict the AM envelopes derived by recursive application of probabilistic amplitude demodulation’

**Comment 9:**

Fig 1. Caption, P5, L2, “..speech excerpts by a neurotypical individuals” > “..excerpts from NT individuals”.

**Response:** We have revised it as follows.

‘speech excerpts from a neurotypical individual’

**Comment 10:**

Fig 1. Caption, P5, L3. “The maximal amplitude is normalized to 0 dB”. For the sake of replicability, please explain how.

**Response:** We thank the reviewer for the pertinent comments. We have explained it in greater detail in the caption of Figure 1 as follows.

‘All speech signals were normalized based on z-scores (i.e., mean = 0, SD = 1)’

**Comment 11:**

P3, Caption Fig 3 and else. What do the error bars represent?

**Response:** Thank you very much for the comment. We stated about the error bars in the captions of Fig 3 and Fig 4 as follows.

‘Error bars indicate the standard deviation of the mean.’

**Comment 12:**

P10, Dynamic interaction, L2, “This suggests that AUT speech and AUT-directed speech share not only......and phonetic rhythms.” Because there are weaker dynamic interactions among the rhythms in AUT speech and AUT-directed speech, they share the same rhythm and have the same interactions? This is too strong a statement, given that there is no analysis to support this.

**Response:** We thank the reviewer for the important comments. This sentence was too strong a statement. We have excluded it from the Discussion section.

**Comment 13:**

P11L10, “This study did not control...between groups” > “sex (gender) differences between groups”. Note that until here, still the reader does not know the respondents’ / questioners’ gender. Given the high predominance of male AUT individuals, one would expect that all were male.

**Response:** We thank the reviewer for the pertinent comment. As indicated, we have revised them in the Discussion section as follows. Further, we have specified the information of gender in the first paragraph of the Results section so that potential readers are aware of these factors before reading the Results and the Discussion sections as follows:

Results

‘Eight adults diagnosed with AUT (all men; age range, 24–44 years) and eight age-matched non-autistic adults (three women, five men; age range, 23–45 years) participated in this study. Six NT and eight AUT respondents were asked by one of two NT questioners (all men) to share their recent experiences on 12 topics. We included 87 samples of AUT-directed speech (from an NT questioner to an AUT respondent), 72 samples of NT-directed speech (from an NT questioner to an NT respondent), 74 samples of AUT speech (from an AUT respondent to an NT questioner), and 55 samples of NT speech (from an NT respondent to an NT questioner).’

Discussion

‘This study, however, did not control for sex differences between groups.’

**Comment 14:**

P11 Limitations, first sentence, “..speech signals are influenced..., could all affect the speech signals. Correct the grammar.

**Response:** Thank you for the comment. We modified the sentence in the Discussion section as follows.

‘The main limitation of this study is that characteristics of speech signals can be influenced by multiple factors other than phonology: frequency, word length, grammatical complexity, and insertions of pauses and fillers.’

**Comment 15:**

P11 Limitations, L8, “Nevertheless, this study used a sufficient sample for the statistical methods”. However, the main problem is that the key samples come from the same two questioners who directed speech to AUT persons.

**Response:** Thank you very much for the important comment. We more clearly stated this important problem in the Limitation section as follows.

‘Another limitation is that although this study used a sufficient sample for the statistical analysis methods (Table 1), and all samples were sufficiently gathered from all speakers in terms of numbers and durations, this study used a small sample size of individuals. Particularly, each respondent was asked by only one of the two NT questioners.’

**Comment 16:**

P12, Data Collection. Here is the first time we know that the NT groups consists of 3 females and 5 males, while the AUT group consists of 8 males. What is the gender of the questioners, who provide the key speech samples? In Limitations (P11) one would at least expect a discussion as to whether results could be interpreted in terms of gender differences in the use of prosody or voice range between males and females.

**Response:** We thank the reviewer for the pertinent comments. The sex of the questioners was male. We have stated this in the first paragraph of the Results section so that potential readers are aware of participant genders before reading the following Results and Discussion sections. Further, as indicated, we have also discussed the limitation in the Discussion section as follows:

‘Six NT and eight AUT respondents were asked by one of two NT questioners (all men) to share their recent experiences on 12 topics.’

‘Further, this study could not control for sex (gender) differences between groups. This suggests that as well as AUT-specific characteristics, participant-specific characteristics may have influenced our findings.’

**Comment 17:**

P12, Data Collection, L3 from bottom, P13L3, P13L4, Caption Fig 5, and possibly elsewhere: “experimental paradigm” / “experiment”. Liberal use of “experiment”. An experiment requires dependent and independent variable(s) and allows inference of causal statements. There was no “experiment” here, there were interviews / talks between people.

**Response:** We thank the reviewer for the pertinent comments. As indicated, we have rephrased it as ‘interview’ throughout the manuscript.

**Comment 18:**

P13L13, “alacrity”. There is probably a more common synonym for this.

**Response:** Thank you for the comment. We have reworded it as ‘agile or prompt’.

**Comment 19:**

P13, last paragraph, L1: “The length of each speech sample was > 10 s. For the sake of replicability of the analysis, the caption of Fig 2 states that there were 4-s excerpts. Were these randomly extracted from the samples of > 10 s? Why, because in this case you could have used samples of at least 4 s, instead of 10 s? Please provide explanation.

**Response:** We thank the reviewer for their comments. The scalogram was used for a qualitative analysis but not for the quantitative analysis (i.e., statistical analysis such as ANOVA, transfer entropy). For scalogram, the CWT was run on each AM envelope from randomly chosen 4-s ‘excerpts’ derived from the total speech signals. This is because a longer scalogram is difficult to qualitatively unveil each level of phonological rhythm hierarchy (i.e., prosody, syllable, and prosody) and the relationship between the different levels of the phonological hierarchy. Based on the qualitative understanding using scalogram, we then quantitatively analysed two types of speech characteristics using three AM envelopes (prosody: 1‒4 Hz, syllable: 4‒12 Hz, phoneme: 12‒40 Hz). They were described in the Methods section as follows:

‘CWT was run on each AM envelope from randomly chosen 4-s excerpts taken from total speech signals (>10 second) of AUT, NT, AUT-directed, and NT-directed speech. Due to the scalogram being longer, it is difficult to qualitatively unveil each level of the phonological rhythm hierarchy (i.e., prosody, syllable, and prosody) and the relationship between the different levels of the phonological hierarchy.’

‘Next, based on the qualitative understanding using scalogram, we decided the frequency index in AM envelopes (prosody: 1‒4 Hz, syllable: 4‒12 Hz, phoneme: 12‒40 Hz), and quantitatively analysed two types of speech characteristics using three AM envelopes.’

**Comment 20:**

P14, Data analysis, last paragraph: “The scalograms...chosen 30-s excerpts of speech. In Fig 2, the x-axis denotes time (30 s)...Now it really gets confusing. Both Fig 2 and S3 (which actually states “S1 Appendix”) show scalograms for 4 seconds. Please provide clear and consistent descriptions.

**Response:** Thank you very much for letting me know of the errors. We corrected them in the Methods section as follows.

CWT was run on each AM envelope from randomly chosen 4-s excerpts taken from total speech signals (>10 second) of AUT, NT, AUT-directed, and NT-directed speech. Due to the scalogram being longer, it is difficult to qualitatively unveil each level of the phonological rhythm hierarchy (i.e., prosody, syllable, and prosody) and the relationship between the different levels of the phonological hierarchy. In Fig 2, the x-axis denotes time (0-4 s), and the y-axis denotes the modulation rate (0.1‒40 Hz).’

**Comment 21:**

Other than the sampling frequency, there is no description of recording equipment, background noise, etc.

**Response:** Thank you very much for the comment. As indicated, we have described the recording equipment, background noise, and so on in the Methods section as follows.

‘During speech recording (Marantz, PMD661MKII MP-REC-002, sampling rate = 44.1 kHz, bit rate = 16 bit) through a condenser-type head-worn microphone (SHURE, model BETA54)…’

‘Interviews were conducted inside a sound-proof room to block background noise.’

**Comment 22:**

P15, 2nd paragraph, “...in case the loudness influenced the spectrotemporal modulation feature”. Four lines below this: “in loudness or sound intensity”. Two lines below this: “in pitch and noise”. Pitch and loudness are psychological constructs, frequency and intensity refer to physics, which is likely meant here.

**Response:** Thank you for the comment. As indicated, we have revised these details in the Methods section as follows:

‘in case the sound intensity influenced the spectrotemporal modulation feature.’

‘AM patterns are implicated in fluctuations in sound intensity’

‘FM patterns reflect fluctuations in spectral frequency’
