# Supplementary material for: Non-autistic persons modulate their speech rhythm while talking to autistic individuals
Source: PLoS One. 2023 Sep 28;18(9):e0285591. doi: 10.1371/journal.pone.0285591 (PMC10538692; doi:10.1371/journal.pone.0285591)
Supplement: Attachment — Submitted filename: Response_to_Reviewer.docx [file pone.0285591.s006.docx]

**RESPONSE TO REVIEWER 1:**

I wish to express our strong appreciation to the Reviewer for the insightful comments on our manuscript. We have studied the Reviewer’s comments very carefully and have made necessary corrections. We feel the comments have helped us significantly improve the manuscript.

**Comment 1:**

Materials and Methods

Authors should state the official name of MSPA, WAIS-III, Wechsler Memory Scale-revised in Materials and Methods.

**Response:** Thank you for the comment. We described the official names in the Methods section as follows.

“the Multi-dimensional Scale for Pervasive Developmental Disorder and Attention Deficit/Hyperactivity Disorder (MSPA) was also used to assess the characteristics of developmental disabilities in general. Furthermore, the Wechsler Adult Intelligence Scale-III (WAIS-III) and Wechsler Memory Scale-revised (WMS-R) were used to measure intellectual level and memory function.”

**Comment 2:**

Authors should cite which version of the Diagnostic and Statistical Manual of Mental Disorders was used.

**Response:** We thank the reviewer for the pertinent comments. We cited the version as follows.

“The AUT diagnosis was made by a psychiatrist according to the Diagnostic and Statistical Manual of Mental Disorders (DSM-5 [26]).”

Reference

“[26] American Psychiatric Association, D., & American Psychiatric Association. (2013). Diagnostic and statistical manual of mental disorders: DSM-5 (Vol. 5, No. 5). Washington, DC: American psychiatric association.”

**Comment 3:**

Authors should add citations to autism spectrum disorder (ASD) characteristics. Specifically, Autism Spectrum Quotient, Adult Self-Report, and Autism Spectrum Screening Questionnaire were used respectively.

**Response:** Thank you for the comment. We cited them as follows.

“the Autism Spectrum Quotient [27], Adult Self-Report [28], and Autism Spectrum Screening Questionnaire [29] were used to measure ASD characteristics.”

Reference

[27] Wakabayashi, A., Tojo, Y., Baron-Cohen, S., & Wheelwright, S. (2004). The Autism-Spectrum Quotient (AQ) Japanese version: evidence from high-functioning clinical group and normal adults. Shinrigaku kenkyu: The Japanese journal of psychology, 75(1), 78-84.

[28] Constantino, J. N., & Gruber, C. P. (2012). Social responsiveness scale: SRS-2 (p. 106). Torrance, CA: Western psychological services.

[29] Ii, T et al.. (2003). 高機能自閉症スペクトラム スクリーニング質問紙 (ASSQ) について. 自閉症と ADHD の子どもたちへの教育支援とアセスメント(English translation: On the high-functioning autism spectrum screening questionnaire (ASSQ). Educational support and assessment for children with autism and ADHD), 39-45. Doi: https://cir.nii.ac.jp/crid/1572261550691517824

**Comment 4:**

It is helpful for readers to understand the characteristics of the participants if the data such as WAIS3, WMS-R, Autism Spectrum Quotient, Adult Self-Report, and Autism Spectrum Screening Questionnaire, Multi-dimensional Scale for Pervasive Developmental Disorder and Attention Deficit/Hyperactivity Disorder are shown in a table or in the text.

**Response:** We thank the reviewer for the pertinent comments.

The publicly available data about characteristics of the participants were summarized into table of Excel spreadsheet and uploaded it to the Open Science Framework for public access (<https://osf.io/ayn2w/?view_only=d87230eaeaea428f94464bf633ae4118>). Additionally, we have minetined this in the Data Availability section as follows.

“The publicly available data about characteristics of the participants were summarized and have been deposited to an external source (<https://osf.io/ayn2w/?view_only=d87230eaeaea428f94464bf633ae4118>).”

Data availability

“All the minimal anonymized data set are available in supporting information (S1, S2, S3 and S4 Appendix) and have been deposited to an external source (<https://osf.io/ayn2w/?view_only=d87230eaeaea428f94464bf633ae4118>).“

**RESPONSE TO REVIEWER 2:**

We are extremely grateful for the Reviewers’ insightful comments on our manuscript. We have studied the Reviewers’ comments carefully and have made the necessary corrections in our paper. We believe that the comments have helped us significantly improve and refine the manuscript.

**Comment 1:**

1. When moving the Materials and Method section to the classic middle position (now from page 6), the authors forgot to fix the Figure order; now Fig. 2 is followed by Fig 6, which should be Fig 3, etc. Captions should be reordered as well.

**Response:** We thank the reviewer for the important comments. As indicated, we corrected the order of all figures in whole manuscript.

**Comment 2:**

Abstract, Line 26, (all men), also L331 (all men). Since there are just two, "..(both men).." might be better.

**Response:** Thank you for the comment. We revised them as follows.

“two NT questioners (both men)”

**Comment 3:**

P6, L147, L148. The acronyms for MSPA, WAIS-III and WMS-R are on P19, L505, 506 and should also be moved to P6.

**Response:** We thank the reviewer for comments. As indicated, we stated these full spells in the Methods section and the acronyms were moved to the Discussion section as follows.

Methods section

“the Multi-dimensional Scale for Pervasive Developmental Disorder and Attention Deficit/Hyperactivity Disorder (MSPA) was also used to assess the characteristics of developmental disabilities in general. Furthermore, the Wechsler Adult Intelligence Scale-III (WAIS-III) and Wechsler Memory Scale-revised (WMS-R) were used to measure intellectual level and memory function.”

Discussion section

“This study also checked the MSPA to control the characteristics of developmental disabilities in general. Furthermore, the WAIS-III and WMS-R were used to control intellectual level and memory function.”

**Comment 4:**

P6, L153, "..about the content of interview.." change to "..the content of the interview.."

**Response:** As indicated, we revised it as follows.

“All participants were informed the content of the interview”

**Comment 5:**

P16, L428, typo "...d14]." change to "..[14]."

**Response:** We corrected it as follows.

“enhancing speech learnability [14].”

**Comment 6:**

Figure 5, x-axis label "NT Questonnair" change to "NT Questioner"

Figure 5, might consider to simplify the axes values "0" and "2" x10-1

**Response:** Thank you for letting me know the typo. We also simplified the axes values "0" and "2" x10-1 to the axes values 0 and 0.2. We revised them as follows.

**Comment 7:**

Figure 7 d, "Onset Rime (12-40 Hz)" (??) change to "Phoneme (12-40 Hz)" as in the main text

**Response:** Thank you for letting me know the typo. We revised it as follows.
